# “It happens to clinicians too”: an Australian prevalence study of intimate partner and family violence against health professionals

**DOI:** 10.1186/s12905-018-0588-y

**Published:** 2018-06-26

**Authors:** Elizabeth McLindon, Cathy Humphreys, Kelsey Hegarty

**Affiliations:** 1 The Department of General Practice, The University of Melbourne, Melbourne, VIC Australia; 2 Department of Social Work, The University of Melbourne, Melbourne, VIC Australia; 3 Royal Women’s Hospital, Victoria, Australia

**Keywords:** Intimate partner violence, Family violence, Violence against women, Sexual assault, Health professionals, Prevalence

## Abstract

**Background:**

The purpose of this study was to measure the prevalence of intimate partner and family violence amongst a population of Australian female nurses, doctors and allied health professionals.

**Methods:**

We conducted a descriptive, cross-sectional survey in a large Australian tertiary maternity hospital with 471 participating female health professionals (45.0% response rate). The primary outcome measures were 12 month and lifetime prevalence of intimate partner violence (Composite Abuse Scale) and family violence.

**Results:**

In the last 12 months, one in ten (43, 11.5%) participants reported intimate partner violence: 4.2% (16) combined physical, emotional and/or sexual abuse; 6.7% (25) emotional abuse and/or harassment; 5.1% (22) were afraid of their partner; and 1.7% (7) had been raped by their partner. Since the age of sixteen, one third (125, 29.7%) of participants reported intimate partner violence: 18.3% (77) had experienced combined physical, emotional and/or sexual abuse; 8.1% (34) emotional abuse and/or harassment; 25.6% (111) had been afraid of their partner; and 12.1% (51) had been raped by their partner. Overall, 45.2% (212) of participants reported violence by a partner and/or family member during their lifetime, with 12.8% (60) reporting both.

**Conclusion:**

Intimate partner and family violence may be common traumas in the lives of female health professionals, and this should be considered in health workplace policies and protocols, as health professionals are increasingly urged to work with patients who have experienced intimate partner and family violence. Implications include the need for workplace manager training, special leave provision, counselling services and other resources for staff.

## Background

Violence against women, specifically intimate partner violence and family violence (hereafter referred to as ‘intimate partner and family violence’, is a serious and prevalent public health issue [1]. Intimate partner violence (IPV) is defined as “any behaviour that causes physical, psychological or sexual harm to those in that relationship” [2], and Family Violence (FV) is defined as harmful behaviour perpetrated by a non-intimate family member at any time in the life course, including the witnessing of violence between parents [3]. Throughout this paper, we use the terms: IPV when referring to violence by a partner, FV when referring to violence by a non-intimate family member, and intimate partner and family violence when referring to both violence by a partner and/or non-intimate family member. Global estimates of IPV are that it affects between 15 to 71% of ever-partnered women across their lifetime [4]. Australia’s IPV prevalence is towards the lower end of that spectrum, with 25% of adult women in a national survey experiencing at least one incident of physical or sexual IPV during adulthood, 2.1% in the last 12 months [5]. The prevalence of physical or sexual violence before the age of fifteen is 16%, predominantly occurring within the family of origin, while 13% of Australian women were exposed to FV as children [6]. IPV contributes to a range of physical, sexual, psychological and reproductive health issues [7]. Survivors of IPV present to health care services more than other women [7], and during pregnancy there are increased risks for the unborn baby [8]. Thus, health professionals are increasingly recognised as having an essential role to play in identifying IPV survivor patients and providing a timely evidence-based response [9]. However, there are barriers to health professionals providing such interventions [10]. These include discomfort discussing the issue, lack of time and knowledge [10], and personal history of IPV [11].

While the majority of nurses and allied health professionals employed at public hospitals are women, little is known about the prevalence of IPV and FV against these health professionals [12, 13]. An extensive search of the academic literature (1987–2017) using three main search terms and synonyms - intimate partner violence, family violence, personal experience and health professional - identified fourteen quantitative studies into intimate partner and family violence against nurses and other health professional groups globally [12, 14–26]. None of these studies were Australian. The lifetime prevalence rate ranged between 3.7% (doctors in the United States) [17] and 97.7% (doctors and nurses in Pakistan) [18]. A large study conducted in a country with a comparable population prevalence rate to Australia is that by Bracken et al. (2010) in the United States [12], who surveyed 1981 nurses and found that the lifetime physical or sexual IPV prevalence rate was 25%. The strengths of this study included the large sample size and response rate (52%); however, this study did not cite the use of a validated scale and asked a small number of IPV questions. More recently, the Cavell Nurses’ Trust surveyed 2254 British nurses about their health and well-being, including their exposure to IPV [26]. They found that, in the last 12 months, 12.2% of nurses had experienced non-physical abuse by a partner, while 3.1% had been physically abused, and these were substantially increased rates to that of the general community [26]. The limitations of the few studies on this issue include: a lack of rigor in the assessment of IPV [12, 14, 16, 17, 20, 22], low or unpublished response rates [20, 23, 26], small sample sizes [21, 23, 24], or publication ten or more years ago [15–17, 24, 25]. Another feature of these studies is their diversity: six of the studies were conducted in countries where a language other than English is the official language [15, 16, 18, 19, 21, 23], and in some countries prevalence studies were hard to generalise to the Australian context since the population prevalence was substantially higher than reported in the Australian community [18, 22, 23].

The primary objective of our study was to address a gap in the available evidence about exposure to 12 month and lifetime IPV and lifetime FV, against female health professionals in Australia. The secondary objectives were to investigate the prevalence of interpersonal violence perpetrated by people other than partners/family members (i.e. colleagues, neighbours, strangers) against health professionals, and to investigate whether age, professional background and/or years of experience were associated with a history of intimate partner and family violence.

## Methods

We developed a cross-sectional survey about health professional’s personal experiences of IPV, FV and other violence in the context of professional clinical care. Our survey included questions about demographics, work-related characteristics, exposure to IPV during the last 12 months and adult lifetime, lifetime FV and lifetime other violence. We defined IPV as physical, sexual and/or psychological violence, including the threat of such violence, occurring within an ‘adult intimate relationship’ (lasting longer than one month) with a partner/boyfriend/girlfriend/husband/wife, since the age of sixteen. We used the Composite Abuse Scale (CAS) to measure the prevalence of IPV; a 30-item well validated self-report measure of physical, sexual and emotionally abusive behaviours [27, 28]. Hegarty et al. (1999) developed the CAS using a sample of 427 Australian nurses (33% response rate) [27]. It measures IPV in the previous twelve months using a 6-point scale, and we adapted it further to measure adult lifetime IPV (since the age of sixteen). The CAS uses cut-off scores, which groups participants into four categories of IPV: ‘Severe Combined Abuse’ (severe physical, emotional and/or sexual violence), ‘Physical Abuse combined with Emotional Abuse and/or Harassment’, ‘Physical Abuse’ alone (not in combination with any other category of abuse), and ‘Emotional Abuse and/or Harassment’ alone (not in combination with any other category of abuse).

We defined FV as encompassing violence directed at a participant by a family member at any time during the life course and/or the witnessing of violence between parents during childhood. Based on a review of the literature, we developed two questions to measure FV; “*Have you ever experienced violence or abuse from a family member? (e.g. someone who is not your partner, like a parent, uncle, in-law, sibling) Yes/No”;* and, *“Growing up, was there ever violence or abuse in your home between your parents? Yes/No”.* While the focus of our study was IPV and FV, we also wanted to capture a participant’s overall experience of interpersonal violence, so we asked one further question about incidents of lifetime violence perpetrated by someone not intimately known, i.e. a patient, colleague or neighbour: “*Have you ever experienced violence or abuse from somebody other than a partner or family member? Yes/No. If yes, please describe”.*

The survey, conducted between 8 August and 31 December 2013, was anonymous and voluntary, and completion implied consent. The research was conducted at a single site – a large, tertiary maternity hospital in Australia. Piloting of the survey led to modifications of the wording. We recruited via two methods: (1) online (Survey Monkey) and (2) a paper-based survey to ensure that health professionals without access to a computer in a confidential setting had the opportunity to participate. A third-party recruiter employed by the hospital administered the survey. The online survey link and encouragement to participate by the Chief Executive Officer were distributed via email to all part-time/permanent clinical staff - nurse/midwives, doctors and allied health professionals. Staff were excluded if they were employed casually, or did not work in a clinical capacity (i.e. administration staff). Two reminder emails were sent, at two and three weeks post recruitment commencement. The third-party recruiter had a de-identified list of identification numbers for all potential participants to ensure that a participant did not submit a survey more than once. Reminder emails were targeted to those who had not yet participated. The third-party recruiter arranged for a paper survey and a reply-paid envelope to be delivered to the timesheet pigeonhole of the remaining eligible health professionals who had not yet participated. Coffee vouchers at the hospital café were offered to all staff as incentive and appreciation for considering participation and were not conditional on having completed the survey. Ethics approval was granted by both the recruiting hospital and the University of Melbourne Human Research and Ethics Committees (Ethics ID: 1339986).

### Statistical analysis

Univariate analyses using frequencies and percentages were performed to describe the sample, including demographics, work-related characteristics and exposure to IPV, FV and other violence. Odds ratios, 95% confidence intervals (CI) and *P-*values were used to assess the likely size of the association between demographic variables and categories of abuse. Data were imported, cleaned, coded and analysed with STATA version 13.

## Results

We sent the survey to 1047 female health professional staff, and 471 participated: 366 completed the survey electronically, while 105 returned a paper version, giving a response rate of 45.0%. The professional background of our sample included: 67.5% (317) nurse/midwives; 14.7% (69) doctors and 13.0% (61) allied health professionals (i.e. social workers, physiotherapists) (Table 1). The participants were very practiced (70.8%, 331 had more than ten years’ experience) and just under half (48.2%, 226) supervised other staff. Participants commonly worked with pregnant women and babies (58.9%, 277 maternity/neonatal services), and were representative of their non-participating peers regarding age, clinical area of work, professional background and years of employment. Most participants (92.9%, 431) had been in an intimate relationship at some time since the age of sixteen.

**Table 1:** Personal characteristics of participating health professionals compared with the research site and the Australian population

Characteristic	No. (%) of participants	% of hospital population ^a^	% of Australian hospital population
Health professional background	(*n* = 470)	(*n* = 1047)	(*n* = 251,000) ^b^
Midwifery	172 (36.6)	51.0 (Nursing and Midwifery)	45 (Nursing and Midwifery)
Nursing	145 (30.9)		
Medical	69 (14.7)	19.2	12
Allied Health	61 (13.0)	10.7	14
Other	23 (4.9)	19.1	**
Age (years)			(*n* = 238,029) ^c^
< 30	81 (17.2)	18.7	15.3
30–39	123 (26.2)	25.9	19.4
40–49	100 (21.3)	21.4	26.0
50–59	133 (28.3)	23.9	28.5
≥ 60	33 (7.0)	10.0	10.7
Intimate relationship status			(*n* = 15,509) ^d^
Current (*n* = 430)	337 (78.4)	n.a.	65
Last 12mths (*n* = 432)	363 (84.0)	n.a.	**
Longer than 12mths (*n* = 464)	431 (92.9)	n.a.	84
Health professional experience (years) (*n* = 468)			
< 5	70 (15.0)	n.a.	**
5–9	67 (14.3)	n.a.	**
10–19	119 (25.4)	n.a.	**
20–29	99 (21.2)	n.a.	**
≥ 30	113 (24.2)	n.a.	**
Employment at hospital (years)			
< 5	197 (42.0)	50.7	**
5–9	104 (22.1)	21.9	**
10–19	94 (20.0)	16.7	**
20–29	61 (13.0)	8.7	**
≥ 30	14 (3.0)	1.9	**

Values are numbers (percentages) unless otherwise stated

Denominators vary due to missing responses

^a^ Comparison female-only hospital data not available. Percentages listed above include male and female health professionals. Proportion of female clinical staff at research site hospital: 88.9%, male: 11.1%

^b^ Australian male and female health professionals [43]

^c^ Australian nurse/midwives [13]

^d^ Population relationship data [44]

n.a. Data not collected by research site

** Comparable data not available

### Twelve-month prevalence of intimate partner violence

More than ten percent of our sample had experienced IPV in the previous 12 months; feeling afraid of a partner was reported by 5.1% (22/432), which when combined with any category of violence on the CAS, increased to 11.5% (50/434) (Table 2). The most common form of violence during the previous 12 months was Emotional Abuse/Harassment alone (6.7%, 25/375), 2.1% (8/375) had experienced Severe Combined Abuse, 2.1% (8/375) reported Physical Abuse and Emotional Abuse/Harassment, and 0.5% (2/375) reported Physical Abuse alone. Rape and/or attempted rape by an intimate partner was disclosed by 1.7% (7/375) of participants.

**Table 2:** 12 month and lifetime prevalence of intimate partner violence. Values are numbers (percentages) unless otherwise stated

Intimate partner violence	12 month	Adult lifetime prevalence
	*n* = 432	*n* = 433
Fear of partner ^a^	22 (5.1)	111 (25.6)
IPV Category (CAS)	*n =* 375	*n* = 421
Severe physical, emotional and/or sexual combined abuse	8 (2.1)	58 (13.8)
Physical abuse and emotional/harassment	8 (2.1)	19 (4.5)
Emotional abuse and/or harassment alone	25 (6.7)	34 (8.1)
Physical abuse alone	2 (0.5)	14 (3.3)
	*n =* 402	*n* = 421
Sexual assault (rape) by partner	7 (1.7)	51 (12.1)
	*n =* 434	*n* = 434
Total: Fear of partner and/or abuse	50 (11.5)	146 (33.6)

Denominators vary due to missing responses

^a^ 33 participants omitted as they had never been in a relationship

### Adult lifetime prevalence of intimate partner violence

One in three (146/434, 33.6%) participants reported fear of a partner and/or scored as having experienced some form of violence since the age of sixteen (Table 2). The most common category of abuse was Severe Combined Abuse, 13.8% (58/421), followed by Emotional Abuse/Harassment alone, 8.1% (34/421), 4.5% (19/421) Physical abuse and Emotional Abuse/Harassment, and 3.3% (14/421) Physical Abuse alone. Around one in ten (51/421, 12.1%) participants had been raped by a partner since the age of sixteen.

### Lifetime prevalence of family violence

The proportion of participants who had experienced violence by a non-intimate family member was 28.4% (133/469) (Table 3). Of this group, 12.8% (60/469) had a combined history of both IPV and FV, and 15.6% (73/469) had survived FV alone with no coexisting history of IPV.

**Table 3:** Lifetime prevalence of intimate partner violence, family violence and other violence. Values are numbers (percentages) unless otherwise stated

Type of violence	Lifetime prevalence
Family violence (including witnessing parental IPV) (*n* = 469)	133 (28.4)
Childhood witnessing parental IPV only	93 (19.8)
Exposure to IPV and/or FV (*n* = 469)	212 (45.2)
Combined history of *both* IPV and FV	60 (12.8)
IPV only	79 (16.8)
FV only	73 (15.6)
Other interpersonal violence (not perpetrated by IP or family) (*n* = 466)	87 (18.7)
Other violence *only* (no combined history of IPV or FV)	26 (5.6)
Total: Lifetime interpersonal violence (by partner, family member and/or other) (*n* = 469)	238 (50.8)

Denominators vary due to missing responses

### Lifetime prevalence of other interpersonal violence

Experiences of interpersonal violence from somebody other than a partner or family member were reported by 18.7% (87/466) of participants (Table 3), and two thirds (70.2%, 61/87) of these participants had a coexisting history of intimate partner and family violence. When asked to describe the type of violence they had experienced, qualitative descriptions were categorised as follows: sexual assault (52.8%, 28/53), physical and/or combined emotional abuse (17.0%, 9/53) and emotional abuse/harassment alone (30.2%, 16/53). The perpetrator of the violence was identified as a stranger by 7.1% (4/56) of participants who provided descriptions of their experience. In contrast, the perpetrators of other interpersonal violence most frequently reported were: friends/acquaintances (29.6%, 16/54), patients (29.6%, 16/54) and colleagues (20.4%, 11/54). Males perpetrated the majority (89.7%) of the other violence described by participants.

### Professional background and violence

We examined whether a participant’s professional background was associated with a history of intimate partner and family violence and found that allied health professionals (65.6%, 40/61) were significantly more likely to report violence by an intimate partner or family member (*p =* 0.001) with increased odds at a level of 2.6 (CI: 1.4–4.6). This was compared to their peers in nursing/midwifery (42.5%, 134/315) and medicine (44.9%, 31/69). Neither age or relationship status was significantly associated with fear or violence during the adult lifetime.

## Discussion

Our study suggests that intimate partner and family violence, including sexual assault, are frequent traumas in the lives of participating women health professionals. One in ten (11.5%) health professionals had felt fear of their partner, or experienced physical, emotional and/or sexual violence from them during the previous 12 months. To put this into context, this is a substantially higher prevalence than the Australian population community sample (2.1%, N= > 17,000) [5], double the prevalence rate identified in a large workplace survey of Australian teachers and nurses (5.0%, *N* = 3611) [29], but lower than a clinical sample of patients in primary care (19.6%, *N* = 1344) [30, 31]. The community surveys have used different methodologies and may not be directly comparable with our survey; however, our findings of a lower prevalence than the clinical sample cited above validates our results, since both samples were assessed using the CAS. We would expect the 12 month rate of IPV to be lower in a sample of currently employed healthcare workers compared with a sample of unwell patients presenting to a primary care doctor with clinical symptoms, since IPV prevalence is consistently higher among those seeking health care, including primary care [32].

Across their adult lifetime, one quarter (25.6%) of participants had experienced fear of a partner, which is similar to the clinical primary care sample discussed above, where the lifetime fear of a partner was 28% (*N* = 1836) [30]. More than one in ten (12.1%) participants had been raped by their partner, which is considerably higher than both the Australian population community sample (9.2%) [5], and a large community sample of women aged 34–39 years where the prevalence (assessed by the CAS) was 6.3% (*N* = 7768) [33]. Forty-five percent of our sample (45.2%) had experienced either violence from a partner or family member, with 12.8% having experienced both. Half (50.8%) of all participants had a lifetime history of interpersonal violence, perpetrated by either a partner, family member or somebody else. These findings are difficult to place in a broader context because of difference in the measures used. They indicate however, that the violence burden in health professional women’s lives may be high and overwhelmingly perpetrated by partners and family members. Further to this finding, while a fifth (19.8%) of participants identified that they had been the victim of violence by somebody outside the home, only a small proportion (5.6%) had experienced this category of violence in isolation; most survivors had a combined history of intimate partner and family violence. When asked about the perpetrator of the other violence, the majority (89.7%) were men known to the survivor: their friends, patients and colleagues.

We found that being an allied health professional was significantly associated with intimate partner and family violence. Since the majority of allied health professionals employed at the research site were social workers, and social workers are regularly referred to once a patient with a history of intimate partner and family violence is identified, they are therefore a professional group who are familiar with discussing narratives of violence [9]. Some research has indicated that people who work in the helping professions may have spent greater time confronting their personal trauma histories motivating them to support others recovering from trauma [34]. It follows then that allied health professionals may have been more willing to disclose intimate partner and family violence in this survey, or they may indeed have a higher prevalence of intimate partner and family violence. Social workers are also at higher risk of experiencing vicarious or secondary trauma from listening to the traumatic stories of their patients [35]. This warns of a potentially high cumulative trauma load stemming from the combination of primary and secondary trauma, and underscores the need for resources to assist health and other helping professionals in their work supporting patients.

Strengths of this study include the well-validated scale we used to measure IPV [27] and the representation of different health professionals. Our study is the first to publish the prevalence of IPV more than 12 months ago using the CAS, another strength of the research. The overall response rate of 45.0% is not optimal, but given the sensitive nature of this survey [36], its length, and the heavy work demands of our participants, it is comparable to similar rigorous research [12]. Other limitations of this study include; self-report and social desirability which might have led to under-reporting of violence, non-response bias and the single recruitment site which prevents generalisability of findings [37, 38]. There is the potential for recall problems with both 12 month, and lifetime measures, or “telescoping”, remembering incidents as occurring more or less than they did [39]. It is also possible that survivors of violence may be more interested and willing to participate in intimate partner and family violence research than other people [38]. These issues acknowledged, over-reporting is widely agreed to be rare in intimate partner and family violence research, while there is substantial concern about underreporting [40]. We found participants more frequently reported Emotional Abuse and/or Harassment in the previous 12 months and Severe Combined Abuse in the period longer than 12 months. Since no evidence suggests that the prevalence of emotional abuse decreases over time, we speculate that the tendency to report non-physically abusive behaviours might recede over time, indicating possible underreporting of lifetime Emotional Abuse and/or Harassment in our study.

## Conclusions

Our study is the first to measure the prevalence of intimate partner and family violence in an Australian health professional population of nurses, doctors and allied health professionals. For the first time, it suggests that intimate partner and family violence may be common in the personal lives of Australian clinicians. These findings have implications for policy, practice and research. Healthcare organisations rarely consider what it means if the health professional is impacted by fear and violence in their home and are asked to intervene sensitively with patients affected by these same issues. Employment can be highly protective for someone experiencing violence [12], but it can simultaneously be a risk [41]. Attendance may be disrupted, as well as one’s capacity while at work [31]. Developing a workplace program that supports health professionals with a trauma history, including their clinical practice with patients experiencing intimate partner and family violence, requires organisational leadership, guidelines for a supportive response and trained individuals to receive disclosures (peer support workers, managers/supervisors, Human Resource staff and Employee Assistance Program professionals) [9]. Workplace programs may be especially necessary given that previous research with social workers has found a greater risk of vicarious trauma in response to working with traumatised patients when the social worker has a history of childhood trauma [42]. More research is required to better understand the needs of health professional women during and after intimate partner and family violence, including the role of the workplace. Intimate partner and family violence not only impacts the health professional survivor herself, we argue that it may have important ramifications for health services’ capacity to provide the best care to patients experiencing the traumatic health sequel of violence. Health services should have safe pathways to care for both health professionals and patients who are experiencing intimate partner and family violence.

## Abbreviations

CAS: Composite abuse scale
FV: Family violence
IPV: Intimate partner violence
